# Transthyretin and Nutritional Status in Critically Ill Adults on Parenteral Nutrition: A Prospective Cohort Study

**DOI:** 10.3390/nu16152448

**Published:** 2024-07-27

**Authors:** Marcela Almeida Linden, Renata Germano Borges de Oliveira Nascimento Freitas, Lidiane Oliveira de Souza Teles, André Moreno Morcillo, Matthew Thomas Ferreira, Roberto José Negrão Nogueira

**Affiliations:** 1 Pediatrics Department, State University of Campinas—UNICAMP, Street Tessália Vieira de Camargo, 126, Barão Geraldo, Campinas 13083-887, SP, Brazil; lidianeoliveiradesouzateles@gmail.com (L.O.d.S.T.); morcillo@fcm.unicamp.br (A.M.M.); 2 Department of Epidemiology, Faculty of Public Health, University of Sao Paulo, Av. Dr. Arnaldo, 715, Cerqueira César, Sao Paulo 01246-904, SP, Brazil; renatagbonfreitas@yahoo.com.br; 3 Faculty of Medicine, University of Sao Paulo, Av. Dr. Arnaldo, 251, Cerqueira César, Sao Paulo 01246-904, SP, Brazil; 4 São Leopoldo Mandic Faculty, Street Dr. José Rocha Junqueira, 13, Pte. Preta, Campinas 13045-755, SP, Brazil; 5 Internal Medicine Department, State University of Campinas—UNICAMP, Street Tessália Vieira de Camargo, 126, Barão Geraldo, Campinas 13083-887, SP, Brazil

**Keywords:** critical patients, nutritional assessment, parenteral support, transthyretin

## Abstract

Background and Aims: Correctly characterizing malnutrition is a challenge. Transthyretin (TTR) rapidly responds to adequate protein intake/infusion, which could be used as a marker to identify malnutrition. Nutritional therapy is used to prevent malnutrition. Parenteral nutrition (PN) requires daily monitoring to determine whether what is being offered is adequate. This article aims to investigate whether the practice of measuring TTR is justified. Methods: Data from patients admitted to the ward or intensive care unit (ICU) were collected at three different times: within the first 72 h (T1) of PN use, on the 7th day (T2), and the 14th day (T3) after the initial assessment. Results: 302 patients were included; the average age was 48.3 years old; the prevalence of death was 22.2%, and 61.6% of the sample were male. TTR values and the effectiveness of nutritional support in these patients were not associated with the outcome; however, meeting caloric needs was related to the outcome (*p* = 0.047). No association was found when TTR values were compared to the nutritional status. Thus, TTR was not a good indicator of nutritional risk or nutritional status in hospitalized patients. Conclusions: Undoubtedly, the TTR measurement was inversely proportional to CRP measurements. It was possible to conclude in this follow-up cohort of hospitalized patients that TTR values were not useful for determining whether the patient was malnourished, predicting death or effectiveness of nutritional support, yet based upon our analyses, a decrease in TTR greater than 0.024 units for every 1 unit increase in CRP might be due to ineffective nutritional supply.

## 1. Introduction

Correctly characterizing malnutrition is challenging. Malnutrition is defined as a change in body composition accompanied by organic dysfunctions that can be observed in chemical tests and clinical evaluations [1]. The prevalence of malnutrition can vary between 20 and 60% of hospitalized patients [2]. This leads to a longer hospital stay, a decrease in response to therapy (such as antineoplastic therapy), major complications, and a greater overall cost of care [3,4]. A study showed that hospital malnutrition, isolated or associated with preexisting diseases, can generate expenses of up to 15.5 billion dollars a year. Thus, the correct nutritional support is fundamental [5], and the first step is an appropriate clinical and laboratory evaluation [2].

Among the laboratory tools is transthyretin (TTR), which responds rapidly to adequate protein intake/infusion. This visceral protein, synthesized in the liver, functions as a blood component that transports thyroxine and retinol [6]. TTR levels can increase by 4 mg/dL per week in patients receiving satisfactory nutritional therapy [1].

Conversely, low TTR levels may be associated with an unfavorable outcome, which can often be attributed to malnutrition [7]. However, inflammation is the most common cause of TTR decrease in hospitalized patients. In fact, the liver prioritizes the production of inflammatory proteins and decreases the production of visceral proteins in this situation [8]. Thus, although TTR is useful in identifying malnutrition, even in the presence of chronic inflammation, when related to acute illness, values should be interpreted with caution [9].

Wang et al. observed that the decline in TTR over one week was an independent predictor of worse prognosis. However, they emphasized that it was not possible to establish whether this prognostic relationship was related to malnutrition, the inflammatory phase or due to a combination of intrinsically linked risk factors [10]. The inflammatory process can lead to a decrease in TTR, which can be considered a nutritional risk factor, as inflamed patients commonly have greater difficulty in eating, leading to malnutrition [8].

Malnutrition can lead to increased morbidity and, therefore, a worsening of the patient’s prognosis and must be minimized or resolved. One strategy used to prevent malnutrition is parenteral nutritional therapy (PN), which is given through the intravenous administration of nutrients either exclusively, when the patient does not have the capacity to use their gastrointestinal tract, or mixed, to complement oral and/or enteral caloric and protein intake, if not sufficient [11]. When planning PN, it is crucial to consider the stage of the disease, the nutritional status, and the caloric and protein needs of each individual to avoid overfeeding [12].

When using PN, the patient must be monitored daily to identify whether what is being offered is suitable for them. Considering that the interpretation of the TTR is still used as a tool for nutritional monitoring, this article intends to investigate whether this practice is justified.

## 2. Materials and Methods

### 2.1. Study Design

A longitudinal prospective study was developed. The total sample consisted of 338 patients, of which 36 were excluded due to not filling out the informed consent form (ICF). There were 302 patients included in the study at the Hospital de Clínicas (HC) of the State University of Campinas (UNICAMP), a quaternary care hospital.

A convenience sample was used. Data were collected at three different times: within the first 72 h (T1) of the beginning of parenteral nutrition (PN), the 7th day (T2), and the 14th day (T3) of the patient’s initial evaluation. This study was approved by the Research Ethics Committee of the Faculty of Medical Sciences at UNICAMP under number 2.676.452.

The inclusion criteria were hospitalization at HC UNICAMP and using PN as their primary source of nutrition. The exclusion criteria were the patient or their legal guardian (in cases of unconscious and pediatric patients) not signing the ICF and those under 18 years of age who could sign the assent form but chose not to do so.

### 2.2. Nutritional Support

In the hospital where the study was carried out, parenteral nutritional support is constantly evaluated by the multidisciplinary nutrition therapy team (MNTT), composed of two doctors specialized in nutrology and parenteral and enteral nutrition, a nurse, a pharmacist, and a nutritionist. The PN prescription is made by one of the doctors and validated by a pre-designed computer program to avoid possible pharmacological incompatibilities. PN was administered as either total parenteral nutrition (TPN) or mixed parenteral nutrition (MPN) (parenteral plus enteral or oral nutrition). The service complies with the recommendations stipulated by ESPEN, ESPGHAN, and ASPEN [13,14,15].

The quantity of calories and protein was calculated by adding all sources of nutrients received (TPN or MPN). In the cases of MPN, the responsible nurse estimated the percentage of oral diet acceptance, as well as enteral nutrition input. This hospital provides standardized protocols for determining oral and enteral nutrition portion sizes. Concerning oral nutrition, the necessary calorie and protein consumption was calculated according to the patient’s estimated consumption. Only the amount of industrialized commercial formula administered was evaluated regarding enteral nutrition since it has pre-defined caloric and protein compositions and concentrations.

### 2.3. Assessment of Nutritional Status

Weight and height were used to calculate the body mass index (BMI) in adults, according to the World Health Organization (WHO) 2000 classification [16]. Weight was measured using a *Plenna Balanças*^®^ digital scale (Sao Paulo, Brazil) with a maximum capacity of 150 kg; height was measured with a vertical metal stadiometer with a capacity ranging from 20 cm to 220 cm. When it was impossible to weigh or measure the patient, the data were obtained from reading the medical record or speaking with the patient or a family member. If the patient was older than six and restricted to the bed, the height was estimated using the formula of Chumlea et al., where the knee height (KH) in centimeters is used in the predetermined equations [17]. For children under six years of age who were confined to bed, height/length was measured using a horizontal wooden stadiometer.

The WHO (2006 and 2007) curves were used to classify the nutritional status of children and adolescents, adopting the Z-score values. The Z-score was calculated using the WHO Anthro version 2.0 and WHO Anthro plus version 1.0.4 software [18].

For the nutritional classification of older adults, those with a BMI between 23 and 28 kg/m^2^ were considered eutrophic. Those below 23 kg/m^2^ were considered malnourished, and those above 28 kg/m^2^ were considered obese [19].

### 2.4. Laboratory Evaluation of the Clinical Picture

Laboratory tests were collected by health professionals responsible for the patient and sent to the Laboratory of Clinical Pathology at HC, where the samples were processed according to the standards required for each test. Nephelometry was used to determine CRP and TTR levels [20].

### 2.5. Assessment of Nutritional Support and Prognosis According to the Evolution of TTR

The evaluation of TTR as a possible biomarker of the effectiveness of nutritional support and the patient’s prognosis was performed according to the adaptation of the algorithm proposed by Delliere and Cynober (2016), which is shown in Table 1 [4].

### 2.6. Statistical Analysis

The normality of the sample variables was evaluated to determine the applicable tests. This established that the Chi-square test or Fisher’s Exact and Fisher–Freeman–Halton tests could be used to assess the association between two qualitative variables. The correlation between TTR values with length of stay and CRP values was evaluated using Spearman’s correlation coefficient.

A multivariate analysis of TTR was performed with predictors CRP, age, and underlying disease. The median TTR was modeled using multivariate quantile regression with a bootstrap. Data were processed using the Statistical Package for the Social Sciences 16.0 software (SPSS Inc., Chicago, IL, USA). The significance level adopted was 5%.

## 3. Results

A total of 302 patients were included in the sample, 61.6% male. 86.1% of the sample were over 19 years old, and the average age was 48.3 years. The inpatient units with the most patients were the clinical and surgical wards, with 68.2% of cases. The use of vasoactive drugs was present in 20.5% of the cases, and mechanical ventilation in 21.5%, while the prevalence of death in the sample was 22.2%, as shown in Table 2.

The reasons for PN indication were bone marrow transplantation (BMT), gastrointestinal tract (GIT) surgeries, including postoperative fistula, abdominal distention of non-surgical origin, metabolic disease, uncontrollable vomiting, and patients with systemic inflammatory response syndrome (SIRS) from multiple causes (collectively referred to as ILEO). The most prevalent cause of death was ILEO; 31.3% of the patients died (*p*-value = 0.015).

Among the underlying diseases, the most frequently observed were polytrauma, hematological and non-hematological neoplasms, organic insufficiencies, previous diseases of the GIT, metabolic syndromes, and cases of acute abdomen sepsis.

The effectiveness of nutritional support was evaluated according to the evolution of TTR in 95 patients; 46 (48.4%) had adequate nutritional support and 49 (51.6%) had inadequate nutritional support. Regarding the prognosis (N = 188), 48 (25.5%) had a good prognosis, 56 (29.8%) had an increased risk of death, and 84 (44.7%) had a higher risk of mortality and length of stay.

TTR values and the effectiveness of nutritional support in the general ward and ICU patients did not show any relationship with respect to outcome; however, meeting caloric needs was related to the outcome (Table 3). No association was found when the TTR values were compared to nutritional status (Table 4). Thus, TTR did not indicate risk or nutritional status in hospitalized patients.

**Figure 1:**
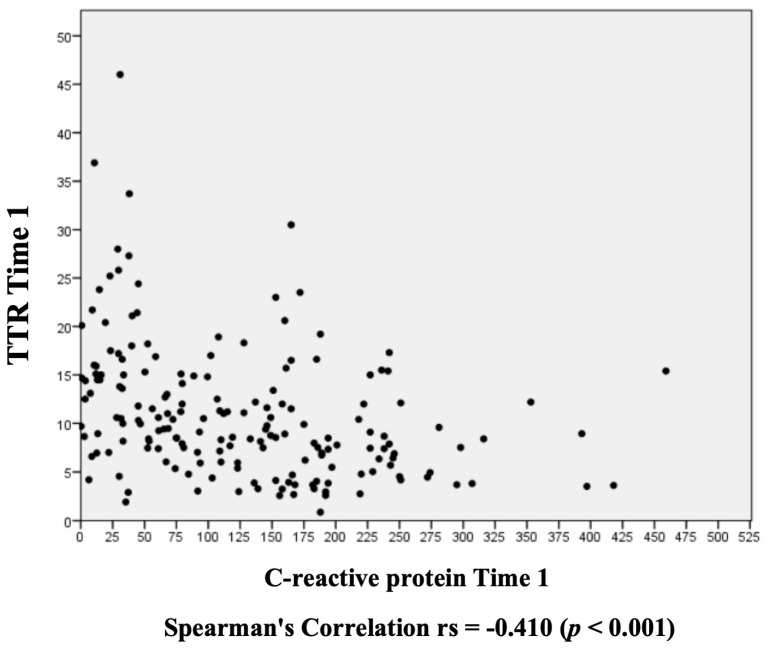
Assessment of the transthyretin and C-reactive protein correlation on day 1.

**Figure 2:**
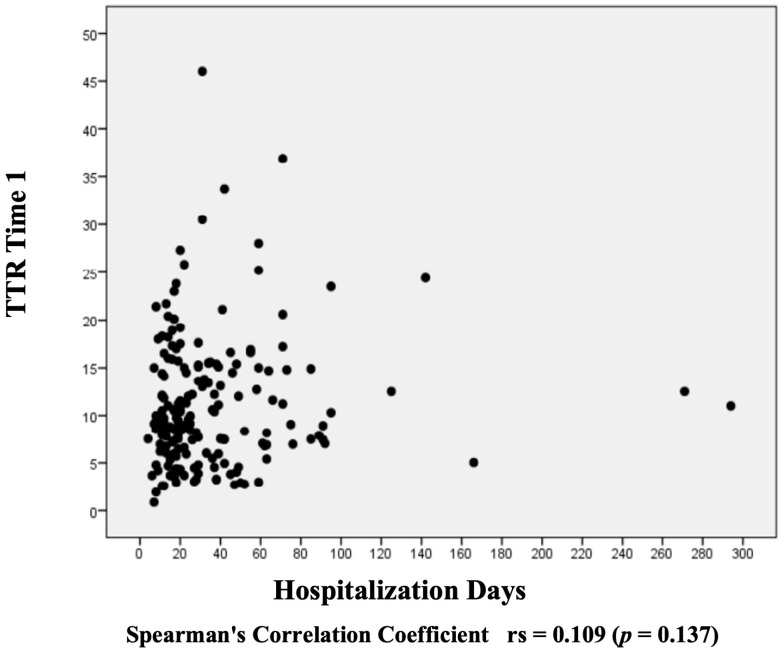
Evaluation of the correlation between transthyretin on day 1 and days of hospitalization.

**Figure 3:**
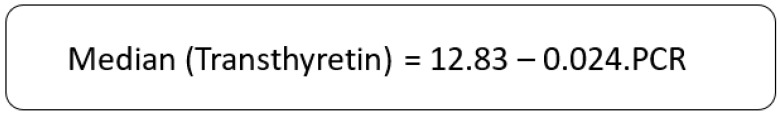
Coefficient of C-reactive protein in relation to transthyretin in the simple model.

The Spearman correlation chart shows a moderate negative correlation between TTR and CRP. The higher the CRP value, the lower the TTR value is (Figure 1). However, no association was found when considering TTR day one values in relation to hospitalization days (Figure 2). In the multivariate analysis, the model demonstrates that the increase in CRP causes a reduction in TTR; for each unit of increase in CRP, there is a reduction of 0.024 units in TTR (Figure 3).

## 4. Discussion

Undoubtedly, the measurement of TTR was closely linked to the dosage of CRP in an inversely proportional way. In this follow-up cohort of hospitalized patients, it was possible to conclude that the TTR values were not useful in determining whether the patient was malnourished. This corroborates what has been previously observed in other studies [8]. In addition, it was not observed as a marker of the possibility of death as believed [1]. What seemed even more controversial is whether TTR could be useful in tracking the effectiveness of patients’ nutritional support. Several previous studies [4] cited TTR measurement as a good marker for this. However, this was not seen in our study or more recent studies [21].

### 4.1. Transthyretin and Mortality

A similar study evaluated the use of TTR in ICU patients using PN; no relationship was found between nutritional intake, mortality, or length of hospital stay in relation to TTR levels. However, the late start of nutritional therapy and the caloric and protein targets only being achieved after the fifth day in the ICU may have influenced the findings, as well as the high level of mortality in the sample (around 45%) [1].

Low TTR values were not associated with the length of stay or mortality in the present study. It is expected that in patients with lower TTR levels—potentially more inflamed—longer hospitalization and/or higher mortality rates would be observed; however, this finding did not occur in this sample. This corroborates the study by Arbab et al., which, despite observing lower TTR values in critically ill patients, found no relationship between TTR, outcome, and days of hospitalization [22]. On the other hand, Issever et al. found that TTR can be an effective biomarker in predicting mortality in patients hospitalized with COVID-19. Patients with lower TTR levels at ICU admission had a higher risk of mortality [23].

Another study observed changes in TTR values associated with mortality in ICU patients, having an inversely proportional relationship. Nutritional support, however, was unrelated to TTR values. In the multivariate analysis, for each 1-point increase in the TTR trend, the chance of death decreased by 6.4%. Thus, the use of TTR as a prognostic indicator is defended, but the clinical relevance still requires further studies, as the relative risks were small [21]. Although our study did not find a relationship with mortality, the regression model demonstrated that the increase of CRP causes a reduction in TTR, where for each unit increase in CRP, there is a reduction of 0.024 units in TTR. This finding can potentially be extrapolated to clinical practice; if the decrease in TTR is greater than this value (0.024 units), one might infer that any additional reduction in TTR could be due to inefficient nutrition.

### 4.2. Transthyretin and Malnutrition

Studies suggest that TTR can be used as a good nutritional marker since its half-life is short (on average 48–72 h) and because it is sensitive to recent food consumption; in other words, it is not influenced by the previous nutritional status, but by the supply of nutrients received by the patient at the time of the exam [4]. However, recent studies propose that TTR values are related to the inflammatory process and not to the nutrition received by the patient [8], as seen in the present study. However, it was observed that the higher the CRP values, the lower the TTR values are. Therefore, TTR is not effective as a nutritional marker; nevertheless, it could indicate that a patient is in an inflammatory state. The inflammatory state can lead the individual to reduce food intake, which would compromise their nutritional status, but most studies that evaluate this relationship, particularly in patients, use PN and/or EN [15]. Therefore, protein dosage for nutritional diagnosis should be performed cautiously and in conjunction with other parameters, and never alone [24].

Malnutrition is known to be associated with longer hospital stays, readmissions, morbid complications, and even a higher mortality rate. Identifying malnutrition early is vital for the proper care of critically ill patients [25]. In our study, no direct relationship was found between nutritional status and mortality; however, it was observed that patients who reached the proposed caloric recommendations had lower mortality rates.

Li et al. clarified that in patients with severe COVID-19—a disease caused by coronavirus 2—the nutritional risk screening (NRS 2002) nutritional assessment score was negatively related to TTR values and positively associated with CD4+ and CD8+ T cell values. Patients screened using the NRS 2002 with low scores and who are treated appropriately may have better clinical outcomes. The authors claim that standard nutritional support can improve cellular immune levels by restoring nutritional status [26].

### 4.3. Transthyretin and Inflammation

In a cohort that evaluated TTR values in kidney transplant patients, it was observed that for each 5 mg/dL reduction in TTR serum levels, there was a subsequent 20% increase in the mortality risk, even after adjusting for confounding factors. Post-transplant patients frequently suffer from inflammation, which causes a decrease in visceral proteins, malnutrition, and poor prognosis. Therefore, monitoring TTR in this population brings benefits in clinical follow-up [27].

More recent studies carried out in severe COVID-19 patients show that the CRP/TTR ratio is adequate to predict the severity of the disease upon admission and during hospitalization. Upon admission, a lower nutritional prognostic index and higher CRP/TTR and CRP/Albumin ratios were related to a higher probability of developing severe COVID-19 [28].

The CRP/TTR ratio was also investigated in patients with cardiovascular disease; the main findings of that study were that the rates of occurrence of death, cardiogenic shock, reinfarction, and acute heart failure were higher in those with a higher CRP/TTR ratio when compared to the group with a lower ratio (*p* < 0.001). Multivariate analysis determined this ratio was an independent predictor for unfavorable outcomes, even after adjusting for confounding factors [29].

Yamada et al. observed that patients with higher CRP levels had lower TTR levels upon admission. Patients with a high CRP/TTR ratio had a higher risk of mortality, whether due to cardiac reasons or not; adjusted multivariate analysis showed that high rates of CRP/TTR ratio upon admission were an independent factor for a higher rate of all-cause mortality in patients with acute myocardial infarction [30].

A systematic review with meta-analysis involving 1813 patients with COVID-19 demonstrated that among 269 patients (14.8%) with the severe form of the disease, lower TTR values are related to the severe development of the disease. They concluded that the progressive decline in TTR reflects a deteriorated clinical status and can be considered a predictor of an unfavorable prognosis [31]. As seen in the Spearman correlation of the study herein, inflammation is related to lower TTR levels, justifying the studies previously cited, which associated low TTR values with the severe form of the disease, characterized by acute inflammation.

As a limitation of this study, we highlight the reduced number of pediatric patients, which made a specific analysis for this population unfeasible. Although the main objective was to follow patients for up to 14 days using PN, most discontinued use before this period due to hospital discharge, suspension of PN, or death. Above all, it is important to emphasize that the data obtained reflect the situation of TTR in an inflamed population. Based on this study, we consider that extrapolation to patients with little or no inflammation cannot be made.

## 5. Conclusions

For patients hospitalized in a high-complexity care hospital, measuring TTR does not bring benefits in terms of nutritional assessment and follow-up. This study corroborates the most recent findings that indicate that the measurement of TTR is an instrument of little value for this purpose in inflamed patients. However, prolonged low TTR values reflect a persistent inflammatory state. However, based on our analyses, a decrease in TTR greater than 0.024 units for every 1 unit increase in CRP might be due to ineffective nutritional supply.

## Figures and Tables

**Table 1:** Interpretation of transthyretin in hospitalized patients [4].

	General Ward	ICU
Is the patient malnourished?	CRP > 15 mg/L—uninterpretable TTR CRP < 15 mg/L—TTR can be interpretable	TTR not interpretable due to level of inflammation—consider nutritional risk
Effectiveness of nutritional support—adequate	TTR increase > 4 mg/dL/week	TTR increase > 4 mg/dL/week
Effectiveness of nutritional support—inadequate	TTR increase < 4 mg/dL/week	TTR increase < 4 mg/dL/week
Prognosis:		
Good prognosis	TTR > 16 mg/dL	TTR > 11 mg/dL
Increased risk	TTR 11–16 mg/dL	-
Increased hospitalization time and mortality	TTR < 11 mg/dL	TTR entre 5–11 mg/dL
Very poor prognosis	-	TTR < 5 mg/dL

Legend: TTR—transthyretin; CRP—C-Reactive Protein; ICU—Intensive Care Unit.

**Table 2:** Characterization of the sample.

Variables (N = 302)	Frequency	Percentage
Sex:		
Female	116	38.4
Male	186	61.6
Age:		
0 to 19 years	42	13.9
Above 19 years	260	86.1
Outcome:		
Discharged	235	77.8
Death	67	22.2
Hospitalization unit:		
ICU	96	31.8
General Ward	206	68.2
Vasoactive drug use:		
Yes	62	20.5
No	240	79.5
Mechanical ventilation use:		
Yes	65	21.5
No	237	78.5
Reason for PN indication:		
GIT Surgery *	187	61.9
BMT **	16	5.3
ILEO ***	99	32.8
Type of nutrition at T1		
Exclusive PN ^#^	178	58.9
PN associated with EN ^##^	36	11.9
PN associated with OR ^###^	75	24.8
PN associated with EN and OR	13	4.3

* TGI—gastrointestinal tract; ** BMT—bone marrow transplantation; *** ILEO—which includes abdominal distension of non-surgical origin–metabolic disturbance, for example, uncontrollable vomiting and systemic infectious response syndrome; ^#^ PN—parenteral nutrition; ^##^ EN—enteral nutrition; ^###^ OR—orally.

**Table 3:** Outcome in relation to transthyretin values and the effectiveness of nutritional support in the general ward or ICU patients.

		Death (N/%)	Discharged (N/%)	*p*-Value *
Transthyretin (N = 207)	At least one TTR result below the reference value	44/23.9	140/76.1	0.817
	All exams with normal TTR	5/21.7	18/78.3	
Effectiveness of nutritional support in general ward patients (N = 68) **	Inadequate	5/15.2	28/84.8	1.0
	Adequate	5/14.3	30/85.7	
Effectiveness of nutritional support in ICU patients (N = 23) **	Inadequate	3/21.4	11/78.6	0.643
	Adequate	3/33.3	6/66.7	
Adequacy of caloric need at T1 (N = 297)				**0.047**
	Inadequate	42/63.6	115/49.8	
	Adequate	24/36.4	116/50.2	
Adequacy of protein need at T1 (N = 298)	Inadequate	46/68.7	148/64.1	0.488
	Adequate	21/31.3	83/35.9	

* Chi-square test probability. ** According to Delliere and Cynober (2016) [4]. Value in bold—statistical relevance.

**Table 4:** Association between nutritional status and transthyretin levels.

	* Nutritional status at time 1		Malnourished	Eutrophic	Obese	Total	*p*-Value **
Transthyretin time 1	Adequate	N %	5 31.3%	5 31.3%	6 37.5%	16 100%	
	Inadequate	N %	50 29.9%	69 41.3%	48 28.7%	167 100%	
	Total	N %	55 30.1%	74 40.4%	54 29.5%	183 100%	0.682
	Nutritional status at time 2		Malnourished	Eutrophic	Obese	Total	*p*-value
Transthyretin time 2	Adequate	N %	1 33.3%	1 33.3%	1 33.3%	3 100%	
	Inadequate	N %	6 35.3%	5 29.4%	6 35.3%	17 100%	
	Total	N %	7 35%	6 30%	7 35%	20 100%	1.0
	Nutritional status at time 3		Malnourished	Eutrophic	Obese	Total	*p*-value
Transthyretin time 3	Adequate	N %	0 0.0%	3 75%	1 25%	4 100%	
	Inadequate	N %	2 66.7%	0 0,0%	1 33.3%	3 100%	
	Total	N %	2 28.6%	3 42,9%	2 28.6%	7 100%	0.141

* Bilateral probability of the Fisher–Freeman–Halton Exact test. ** Nutritional classification according to WHO 2000 for adults and WHO 2006 and 2007 for children.

## Data Availability

The data are available at the State University of Campinas Medical School in the Department of Pediatrics.
